# Effectiveness of Counseling for Infertile Couples on Women's Emotional Disturbance: A Randomized Clinical Trial

**DOI:** 10.1055/s-0041-1736305

**Published:** 2021-12-06

**Authors:** Tayebeh Mokhtari Sorkhani, Atefeh Ahmadi, Moghaddameh Mirzaee, Victoria Habibzadeh, Katayoun Alidousti

**Affiliations:** 1 Department of Midwifery, School of Nursing and Midwifery, Kerman University of Medical Sciences, Kerman, Iran; 2 Department of Biostatistics and Epidemiology, School of Public Health, Kerman University of Medical Sciences, Kerman, Iran; 3 Department of Obstetrics and Gynecology, Afzalipour Clinical Center for Infertility, Kerman University of Medical Sciences, Afzalipour Hospital, Kerman, Iran

**Keywords:** infertility, emotion, social support, anxiety, depression, counselling, infertilidade, emoção, apoio social, ansiedade, depressão, aconselhamento

## Abstract

**Objective** The psychosocial burden of infertility among couples can be one of the most important reasons for women's emotional disturbance. The goal of the present study was to investigate the effect of counseling on different emotional aspects of infertile women.

**Methods** The present randomized clinical trial was performed on 60 couples with primary infertility who were referred for treatment for the first time and did not receive psychiatric or psychological treatment. Samples were allocated to an intervention group (30 couples) and a control group (30 couples) by simple randomization. The intervention group received infertility counseling for 6 45-minute sessions twice a week, and the control group received routine care. The Screening on Distress in Fertility Treatment (SCREENIVF) questionnaire was completed before and after the intervention. Samples were collected from November to December 2016 for 3 months. For the data analysis, we used the Statistical Package for the Social Sciences (IBM SPSS Statistics for Windows, IBM Corp., Armonk, NY, United States) software, version 19.0, and the paired *t* -test, the independent *t* -test, the Mann-Whitney test, the Wilcoxon test, and the Chi-squared test.

**Results** The mean age of the participants was 33.39 ± 5.67 years. All studied couples had primary infertility and no children. The mean duration of the couples' infertility was 3 years. There was a significant difference regarding depression (1.55 ± 1.92; *p* < 0.0001), social support (15.73 ± 3.41; *p* < 0.0001), and cognitions regarding domains of fertility problems (26.48 ± 3.05; *p* = 0.001) between the 2 groups after the intervention, but there was no significant difference regarding anxiety (25.03 ± 3.09; *p* = 0.35).

**Conclusion** The findings showed that infertility counseling did not affect the total score of infertile women' emotional status, but improved the domains of it except, anxiety.

## Resumo

**Objetivo** A carga psicossocial da infertilidade entre casais pode ser uma das razões mais importantes para os distúrbios emocionais em mulheres. O objetivo deste estudo foi investigar o efeito do aconselhamento em diferentes aspectos emocionais de mulheres inférteis.

**Métodos** Este ensaio clínico randomizado foi realizado com 60 casais com infertilidade primária indicados para tratamento pela primeira vez mas que não receberam tratamento psiquiátrico ou psicológico. As amostras foram alocadas em um grupo de intervenção (30 casais) e um grupo de controle (30 casais) por randomização simples. O grupo de intervenção recebeu aconselhamento sobre infertilidade por 6 sessões de 45 minutos 2 vezes por semana e o grupo controle recebeu cuidados de rotina. O questionário de Triagem por Aflição no Tratamento da Infertilidade (Screening on Distress in Fertility Treatment SCREENIVF em inglês) foi preenchido antes e após a intervenção. As amostras foram coletadas de novembro a dezembro de 2016 durante 3 meses. Para a análise dos dados usamos o programa Statistical Package for the Social Sciences (IBM SPSS Statistics for Windows IBM Corp. Armonk NY Estados Unidos) versão 19.0 e os testes- *t* pareado e independente e os testes de Mann-Whitney de Wilcoxon e do qui quadrado.

**Resultados** A média de idade dos participantes foi de 33 39 ± 5 67 anos. Todos os casais estudados tinham infertilidade primária e não tinham filhos. A duração média da infertilidade dos casais era de 3 anos. Houve diferença significativa quanto à depressão (1 55 ± 1 92; *p* < 0 0001) ao apoio social (15 73 ± 3 41; *p* <0 0001) e às cognições em relação aos campos dos problemas de fertilidade (26 48 ± 3 05; *p* = 0 001) entre dois grupos após a intervenção mas não houve diferença significativa com relação à ansiedade (25 03 ± 3 09; *p* = 0 35).

**Conclusão** Os achados mostraram que o aconselhamento sobre infertilidade não afetou a pontuação total do estado emocional de mulheres inférteis mas melhorou seus campos exceto a ansiedade.

## Introduction

Infertility is one of the major problems in gynecology, and is defined as the inability to become pregnant with regular sexual intercourse for more than one year without the use of birth control methods. 1 2 It has been estimated that 8% to 12% of couples worldwide, 3 and 11.2% to 14.1% of Iranian couples, 4 have experienced infertility. The inability to become pregnant profoundly affects the various aspects of the lives and relationships of infertile couples. 5 6

Infertile couples may be at risk of developing mental health problems. 7 Impulsive behavior, anger and frustration, anxiety, loss of control over sexual feelings, and vulnerability are observed among infertile individuals. 1 2 The prevalence of psychological problems among them is between 25% and 60%. The incidence of depression and anxiety in these individuals is significantly higher than in fertile individuals and the general population. 8

Studies 9 10 have shown that couples who underwent in-vitro-fertilization (IVF) treatments showed more emotional and anxiety disorders and had lower scores in psychological areas than controls. In a study by Huppelschoten et al. (2013), 11 infertile women and their sexual partners needed appropriate and adequate psychological support at all stages of treatment. Ashraf et al. (2014) 12 found that infertility reduces the various aspects of quality of life of infertile women. Mosalanejad et al. (2012) 13 showed that cognitive-behavioral therapy (CBT) reduces the stress of infertile women and increases the adaptation to mental problems caused by infertility. Soltani et al. (2014) 14 also concluded that focused emotional therapy can reduce depression, anxiety, and stress in infertile couples, and Faramarzi et al. (2013) 15 found that CBT could be a reliable alternative to fluoxetine to reduce the stress caused by infertility. 14 15

Several studies 1 14 have shown that emotionally-focused therapy can decrease the amount of depression, anxiety, and stress in infertile couples. Infertility counseling is also a special approach to manage emotional problems associated with infertility, and it is a guide for husbands and wives to relieve their anxiety, hatred, anger, and dissatisfaction. 1 16 The goal of infertility counseling is to help couples develop successful coping strategies to deal with the short- and long-term outcomes of infertility, resolving and understanding the issues related to it, and finding a more satisfying lifestyle. 17 18 The chances of success with assisted reproductive techniques are higher when the mental state of couples is in balance. To moderate the mental state, drug interventions may interfere with assisted reproductive techniques or require regular and long-term use. Consultation and psychotherapy are non-medical interventions that can reduce the negative effects of depression and emotional problems. 19 Different studies 14 20 found that couples therapy can be effective for the mental health of women with biopsychosocial problems. In addition, most counselling interventions require the participation of the patients for several sessions. Regarding the condition of our population and location of our psychosocial program, planning for the minimum number of sessions was necessary. Therefore, infertility counselling was adopted based on international guidelines with an eclectic approach to the women's biopsychosocial issues regarding the couple's infertility. 9

To increase the chances of fertility and reduce mental conflicts, given the high prevalence of infertility, the lack of psychosocial support, and insufficient scientific information provided to infertile women, family counseling in the management of infertile couples seems critical. 21 The present study was conducted to investigate the effect of counseling on different emotional aspects of infertile women.

## Methods

### Study Design

The present randomized clinical trial was designed to investigate the effect of counseling on different emotional aspects of infertile women referred to Afzalipour Hospital Infertility Center in Kerman, Iran, from October to December 2016. Afzalipour Hospital Infertility Center is the only public center that offers special services for infertility in Kerman province. Couples who were willing to participate in the study and met the inclusion criteria were included.

We included in the study couples with primary infertility (regardless of the cause) who were referred for treatment for the first time and did not receive psychiatric or psychological treatment. The exclusion criteria were the spontaneous occurrence of pregnancy during the counseling sessions, or couples who missed two out of the six counseling sessions.

**Fig. 1:**
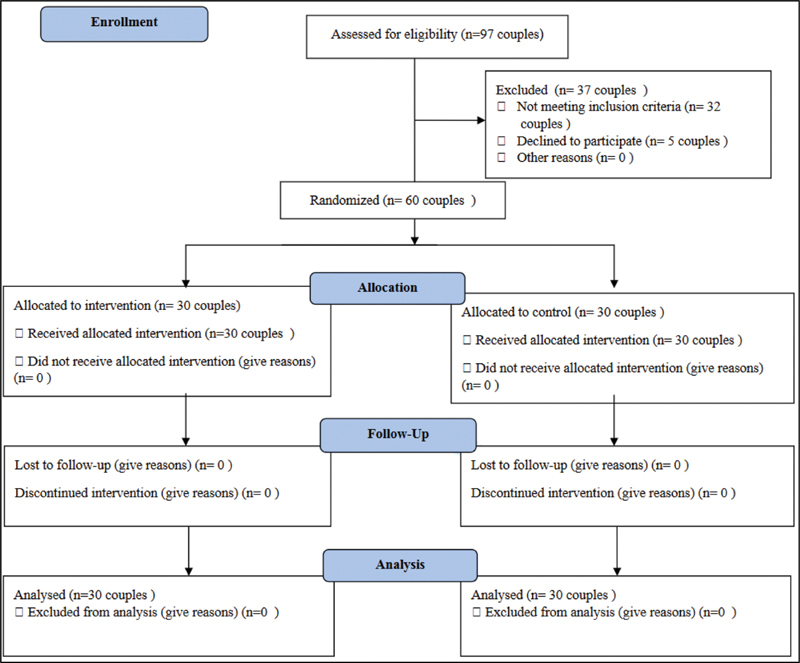
CONSORT Flowchart of the selection of participants for the study.

According to a previous study 22 with a type II error of 20%, and according to



the sample size was estimated as 60 couples. it means 30 couples in each control and intervention group. They were selected based on convenience sampling, and were randomly allocated to the intervention and control groups. During the intervention, three couples were excluded because they did not attend all the meetings, and two couples were excluded due to the absence of the husbands. In the following counseling group, new couples who met the criteria were included in the study to replace those excluded. In the control group, there were no dropouts during the study ( Fig. 1 ).

### Instruments

We used multiple questionnaires. The demographics questionnaire contained questions regarding age, gender, level of schooling, time since contraceptive discontinuation, number of children, length of contraceptive use, type of birth control method, causes of infertility (female or male), and history of psychiatric problems before infertility.

The Screening on Distress in Fertility Treatment (SCREENIVF) questionnaire was developed by Verhaak et al. (2005), 23 24 and it contains 34 items divided into 4 subscales that include state anxiety, trait anxiety, depression, social support, and cognitions regarding fertility problems. Anxiety was assessed with questions 1through 10 of the Spielberg Questionnaire. Depression was assessed with questions 11through 17 of the Beck Depression Inventory. Social support was assessed with questions 18 through 22 of the Social Participation Questionnaire. Failure to accept infertility problems was assessed with questions 23 through 28, and acceptance of infertility problems were assessed with questions 29 through 34, of the IVF Patient Recognition Questionnaire. The cut-off scores were as follows: depression – ≤ 4; anxiety – ≤ 24; cognitions regarding fertility problems – ≤ 14; and social support – ≤ 15. All dimensions have a high degree of reliability. The Cronbach alpha (α) for depression, anxiety, helplessness, and cognitions regarding fertility problems were of 0.82, 0.88, 0.87, 0.92, and 0.89 respectively. 25 26 27 The SCREENIVF correctly identified 69% of the total of patients who presented clinically-significant emotional difficulties and 77% of those who did not. The original version of the SCREENIVF showed excellent reliability in all scales (Cronbach α between 0.82 and 0.92). 28

To compose the Persian version of the SCREENIVF, we performed forward and backward translations of the validated Portuguese version of the questionnaire, which showed reliability in all dimensions (Cronbach α ≥ 0.70, except depression among men: α = 0.66). 27 The reliability of the translated questionnaire was confirmed through the application of a pretest with the participation of 50 people and a posttest after 2 weeks, with a Cronbach α of 0.7 and an intraclass correlation coefficient (ICC) of 0.74.

### Procedures

The present study was approved by the local Ethics Committee of Kerman Medical University (IR.KMU.REC.1395.678IR). After obtaining the necessary permissions from the head of Afzalipour Hospital Infertility Center, the researcher invited all infertile couples who met the inclusion criteria to participate in the study, which was conducted from October to December 2016.

The couples were assigned to the intervention or control groups according to the day of referral to the infertility center. In the even days of the first week, they were assigned to the intervention group, and, in the odd days, they were assigned the control group, and the opposite was done in the following week.

The researcher explained the purpose of the study to the participants and obtained written informed consent. The couples were also assured that the collected information was confidential and would only be used for research purposes. Then, they were asked to answer the questionnaire for the pretest. There was no blinding in the research process.

Each of 6 counseling sessions, lasting 45 minutes, were held in a suitable room at the Infertility Center and conducted by a trained midwife.

The infertility group counseling, which was based on guidelines issued by Boivin and Kentenich, 9 was composed of a combination of psychological training, supportive counseling, and cognitive-behavioral counseling. The content of the infertility counseling sessions is shown in chart 1 .

**Chart 1:** Content of the infertility counseling sessions

Session	Content
One	Outlining sessions' goals, introducing female and male genital system and fertility mechanism, and causes of infertility
Two	Acquaintance with the benefits and side effects of assistaned reproductive therapies (ART),
Three	Acquaintance with mental disorders caused by infertility problems
Four	Acquaintance with all possible support systems during infertility
Five	Training enriching relationships, relaxation, and coping techniques
Six	Decision-making about therapy continuation, discontinuation, and replacement of options

The control group received routine care, and after post test, the counseling sessions were held for them. At the end of the last session, the posttest was applied both groups. The pre- and posttest questionnaires were filled out by the researcher. 29 30

### Ethical Considerations

The present article is the result of a dissertation approved by Kerman University of Medical Sciences under the code of ethics number IR.KMU.REC.1395.678 and the clinical trial code number IRCT2017080124866N4, and it was supported by the research deputy of Kerman University of Medical Sciences. The purpose of the study was explained to the subjects, and they were included after signing the written informed consent.

### Statistical Analysis

Data were analyzed using the Statistical Package for the Social Sciences (IBM SPSS Statistics for Windows, IBM Corp., Armonk, NY, United States) software, version 19.0. Descriptive statistics (frequency, percentage, mean, and standard deviation) were used to detail the characteristics of the sample. The Chi-squarde test was used to determine the consistency of the two groups in terms of demographic variables. If parametric conditions were present (normal distribution and equality of variances), parametric statistical tests (dependent *t* -test for the comparison of the groups and paired *t* -test for the comparison of the groups before and after the test) were used. Otherwise, the non-parametric equivalents (Mann-Whitney and Wilcoxon tests) were used. Values of *p* < 0.05 were considered statistically significant.

## Results

The mean age of the participants was 33.39 ± 5.67 years, and the level of schooling of most of them was incomplete high school among the intervention group, and complete high school among the control group. Most female participants were housewives in both groups. All studied couples had primary infertility and no children. The mean duration of the infertility was three years. On average, couples used various birth-control methods for 12.01 ± 4.58 months. Regarding the infertile subjects in both groups, men were the majority (34.15%). None of the subjects reported history of psychiatric diseases (depression, obsession, or anxiety) before they became aware of the infertility. Based on the Chi-squared test, there were no significant differences among the sample in terms of gender, age, and level of schooling at the time of inclusion in the study, so the two groups were homogeneous regarding demographics ( Table 1 ).

**Table 1:** Demographics of the study sample

VariablesIntervention group – n (%)		Control group –n (%)	*p* -value
**Age (years)** 20–29 30–39 40–50	20 (33.3)	15 (25.0)	0.15
	33 (55.0)	32 (53.3)	
	7 (11.7)	13 (21.7)	
**Gender** Female Male	30 (50)	30 (50)	1.00
	30 (50)	30 (50)	
**Level of schooling** Incomplete high school Complete high school Complete higher education	24 (40)	18 (30)	0.36
	17 (28.3)	25 (41.7)	
	19 (31.7)	17(28.3)	
**Infertile subject** Man Woman Both Unknown cause of infertility	20 (33.3)	21 (35)	0.45
	20 (33.3)	19 (31.7)	
	13 (21.7)	11 (18.3)	
	7 (11.7)	9 (15)	

Different dimensions of the emotional status of infertile women were compared between two groups. The decrease in the depression score after the counseling in the intervention group was statistically significant ( *p* ≤ 0.0001). There was a significant increase in the mean score for social support after the counseling in the intervention group ( *p* ≤ 0.0001). The results showed that counseling had an effect on the the mean scores of intervention group for cognitions regarding fertility problems ( *p* = 0.001). The changes in the mean scores for anxiety were not significant ( *p* = 0.35) after counselling. The differences between thegroups are shown in Table 2 . There were no statistically significant differences between the groups regarding the total score for the emotional status ( *p* = 0.47).

**Table 2:** Mean scores on the subscales of emotional satus for the two study groups

Subscale	Intervention group	Control group	*p* -value *
Anxiety	25.03 ± 3.09	24.58 ± 3.72	0.41
Depression	1.55 ± 1.92	4.46 ± 4.13	> 0.0001
Social support	15.73 ± 3.41	13.43 ± 3.01	< 0.0001
Cognitions regarding fertility difficulties	26.48 ± 3.05	25.40 ± 4.11	0.38
Total score	68.80 ± 6.38	7.66 ± 67.88	0.47 *

* Paired T-Test.

## Discussion

Infertility counselling had different effects on different dimensions of the emotional status of infertile women. A comparison regarding depression showed that infertility counseling was able to reduce the level of depression. Karami et al. (2018) 31 reported that muscle relaxation training had an impact on depression, anxiety, and stress, and Talaei et al. (2014) 32 reported that cognitive behavioral therapy was effective in decreasing depression and psychological problems, among infertile women. The reason for this consistency in results could be the provision of similar counseling sessions in the present and in other studies 26 31 Many studies 6 have reported the negative effects of infertility on marital adjustment and satisfaction, depression, and quality of marital life. Counseling helps couples process their feelings and reach a comfortable state; as a result, their quality of life increases and leads to a decrease in the levels of depression. 26

In the intervention group, the mean scores after counseling regarding the social support subscale were significantly different from those of the control group. The results of the present study were consistent with those of the study of Adl (2016), 33 a quasi-experiment on “the effectiveness of group psychotherapy based on the quality of life and perceived social support in infertile women”. Social support is defined as the perceived level of kindness, companionship and attention received from family members, friends and others. The main function of the perceived social support is that the person feels they are respected and part of a network of mutual duties. 34 One of the main goals of infertility counseling is that patients understand their therapeutic choices and receive enough emotional support to cope with the outcomes of infertility. This empowerment increases their satisfaction and decreases the incidence of negative reactions. 1

Regarding the anxiety subscale, the results indicated that counseling had no effect on infertile women, which is in disagreement with the results of other studies, such as those on the effect of acceptance and commitment therapy, by Rahimi et al. (2018), 35 and on the use of acupuncture, by Hassanzadeh Bashtian et al. (2016). 36

Although group infertility counseling was conducted for couples during 6 sessions lasting it seems that the lack of suitable accommodations for non-resident patients in the city, and the economic problems faced by young people could be the reason for the ineffectiveness of counseling on general anxiety. Also, since it was participants' first time at our specialized center, it seems that the lack of familiarity with it, as well as with the medical staff and the therapeutic processes, and their concerns about the possible outcomes also affected their levels of anxiety.

As for the subscale of cognitions regarding fertility problems, counseling was effective, which is in agreement with the results of the study by Kheirkhah et al. (2014), 37 who reported that group counseling was effective in helping the subjects adapt to infertility. Perhaps the reason for this consistency was the provision of infertility psychological counseling by a trained researcher which was similar in both studies. Psychotherapy and counseling are effective in reducing many psychological problems, improving the quality of life, and they help couples accept the reality of their lives and face the upcoming challenges. 26

The results of the present study are not consistent with those of recent studies. Loucks (2015), 38 in a study on “group therapy as a social response to infertility”, reported that the treatment had an effect on the emotional status of couples. Soltani et al. (2014), 14 in a quasi-experiment, reported that emotionally focused therapy had a positive effect on the couples' emotional distress. In a randomized clinical trial, Mosalanejad et al. (2012) 13 reported that CBT with emotional disclosure had an effect on the mental health status of infertile women. The reason for this inconsistency could be the different sample sizes, the type of intervention, and the differences in the gender distribution of the sample. In the study by Loucks 38 , which was conducted with interviews, there was a potential for a positive bias. In the study by Soltani et al. 14 , the intervention was only conducted among women. It should be noted that women can be more influenced than men, and starting the infertility treatment usually takes time, and affects the emotional status of women, and makes them more anxious, so he conducted his study only on women. 14

The present study was conducted with couples. Since the emotional issues are deep, they require more detailed counseling and a lot more time. One of the reasons that counseling is not effective in improving the score for the overall emotional status is that the number of training sessions is low in proportion to the depth of the problem. Also, due to the subjects' age and their emotional development and stability at this age, it is expected that changes will not easily occur and cannot be easily addressed during the counseling sessions. Furthermore, because of the existing culture and the influence and interference of families in terms of fertility and infertility, it seems that education and counseling to couples are not enough to change their emotions.

## Conclusion

The findings of the present study show that infertility counseling did not affect the total score for the emotional status of infertile couples, but improved the cognitions regarding fertility problems, social support, and depression, and did not improve the levels of anxiety. According to the results, counseling is one of the ways of improving the psychological state of infertile couples. We suggest that, from the time of diagnosis and initiation of treatment, coherent and regular planning should be considered to provide more comprehensive education and counseling to improve all aspects of the emotional status.
